# Advancing space medicine: a global perspective on in-orbit research and future directions

**DOI:** 10.1186/s40779-024-00587-8

**Published:** 2025-01-03

**Authors:** Shan-Guang Chen, Xiao-Ping Chen, Bin Wu

**Affiliations:** 1 National Key Laboratory of Human Factors Engineering, China Astronaut Research and Training Center, Beijing, 100094 China; 2 State Key Laboratory of Space Medicine, China Astronaut Research and Training Center, Beijing, 100094 China

**Keywords:** Space medicine, International Space Station, In-orbit experiments

As humanity ventures deeper into space, our challenges become increasingly complex. Space medicine, once confined to ensuring the health and safety of astronauts on low-Earth orbit missions, is now tasked with ensuring the health and safety of astronauts embarking on extended missions to the Moon, Mars, and beyond. The advancement of space medicine and the conduct of in-orbit medical experiments not only determine the boundaries of human exploration of the cosmos but also provide new insights that can benefit human health on Earth.

## International advances in space medicine: in-orbit experiments

Recent study aboard the International Space Station (ISS) has provided invaluable data on various aspects of astronaut health, including bone density loss, muscle atrophy, and cardiovascular changes [1]. The National Aeronautics and Space Administration (NASA) Twins Study, which compared the health of the astronaut Scott Kelly, who spent nearly one year in space, with that of his identical twin brother Mark Kelly, who remained on Earth, highlighted the significant changes that occur in the human body during extended spaceflight [2]. Research findings on the telomere length, gene expression, and immune response have improved our understanding of how spaceflight impacts the human body at the molecular level [2].

On the basis of these insights, the recent publication of “The Space Omics and Medical Atlas (SOMA) and international astronaut biobank” in *Nature* represents a significant milestone in space medicine [1]. SOMA integrates data from the NASA Twins Study and other international research, offering the most comprehensive mapping of the molecular and physiological effects of space travel to date. The collaboration of over 100 institutions across more than 25 countries aims to advance our understanding of the effects of spaceflight on human health. A battery of cutting-edge technologies, such as single-cell sequencing, spatial transcriptomics and cell-free RNA profiling, was utilized to analyze the biospecimen samples collected from Inspiration4 crew members and generated a more than tenfold increase in human space omics data. Artificial intelligence (AI) and machine learning algorithms have also been integrated to analyze these data and predict health trends or potential issues. Another key contribution of SOMA is the establishment of an international astronaut biobank, which stores viable frozen samples for future analyses, ensuring the sustainability and expandability of this scientific endeavor [3]. Moreover, the accessibility of SOMA data and samples in the astronaut biobank fully reflects the collaborative spirit of scientific research, ensuring that a wider range of experts can contribute to the field, promote innovation, and accelerate the pace of discovery. Overall, the successful implementation of the SOMA plan demonstrates the effectiveness of organized scientific research in advancing our knowledge and capabilities in space medicine, and highlights the importance of international cooperation in addressing complex scientific challenges.

## China’s progress in space medicine: in-orbit experiments

By the end of 2022, the Chinese Space Station (CSS) had been fully deployed and entered the application and development phase. With great success in human spaceflight, China has made significant progress in the field of space medicine research [4]. In the past two years, 5 Shenzhou manned spacecraft (SZ-14, -15, -16, -17, and -18) with 15 astronauts have been on board the station, demonstrating China’s remarkable ability to provide astronauts with comprehensive protection against the physiological effects of weightlessness during long-duration (180-day) spaceflights [5].

An advanced and highly integrated space experiment platform has been constructed. In-orbit space medicine research mainly includes study on cardiovascular function, bone formation, muscle adaptation and gait analysis, brain functional, eye-hand coordination, mental fatigue, biological rhythm monitoring, and visual function. Specifically, the SZ-15 crew aboard China’s Tiangong space station has achieved a world-first accomplishment by successfully capturing three-dimensional (3D) images of their skin cells using a domestically developed two-photon microscope. The microscope’s submicron resolution provides noninvasive microscopic imaging that clearly shows the 3D distribution of the astronauts’ skin structure and cells, while it allows examination of the metabolic stress response of intracellular mitochondria, which will aid astronaut health monitoring in orbit. Recently, one study from the CSS revealed that microgravity impairs the utilization of thiamine in human pluripotent stem cell-derived cardiomyocytes, which in turn disrupts the tricarboxylic acid cycle, leading to cytoskeletal remodeling and an imbalance in calcium homeostasis [6]. Additionally, research on the application of traditional Chinese medicine (TCM) in space has also been conducted, including studies of TCM diagnostic methods, portable acupoint stimulation, and the development of Chinese space fitness exercises. Human factors research and space neuroscience have been implemented to ensure the well-being of astronauts and safeguard their physical and mental health in orbit. These advancements in space medicine are crucial for the sustainable development of China’s manned space program and provide a solid foundation for the implementation of future space missions.

## Future directions for China’s space medicine

Undoubtedly, the SOMA marked a monumental advancement in the field of international space medicine. However, astronauts embarking on upcoming manned lunar missions and future Mars exploration missions face much more complicated health risks, including: (1) Space radiation. Exposure to space radiation is a significant concern, as it can lead to an increased risk of cancer, alterations in the gut microbiome, accelerated atherosclerosis, bone remodeling, and hematopoietic effects. (2) Microgravity. Prolonged exposure to microgravity affects every organ system and can result in an increased likelihood of acute life-threatening events, which requires the immediate management of medical emergencies in situ with real-time guidance from Earth-based physicians. Under some extreme conditions, the ability to provide surgical interventions is necessary because of the impracticality of evacuating to Earth for medical treatment. (3) Mental health. Confinement, isolation, and a lack of social interaction during long-duration space travel can lead to mental health disturbances such as depression and anxiety. Notably, the high-pressure environment of space missions and the responsibility for mission success can also impose additional psychological stress on astronauts. Therefore, psychological support is indispensable for astronauts to maintain their mental well-being and enhance their performance and decision-making capabilities. (4) Infection risk. The risk of infection is a concern, especially for bacteria that may become more resistant to antibiotics in the space environment. All these challenges have set higher standards for the development of China’s space station and advancements in space medicine.

First, the CSS offers a robust platform for organized scientific research. The China Manned Space Agency and the United Nations Office for Outer Space Affairs have selected 9 projects from 17 countries for the initial round of international cooperation. For the next 10 years, more collaboration with scientists worldwide is needed to foster international partnerships by offering opportunities for joint research, technology exchange, and knowledge sharing among participating countries.

Second, more cutting-edge technologies should be integrated to deepen our understanding of the impact of spaceflight on humans and enhance astronauts’ health and performance during space missions. For example, AI tools and big data analytics have great potential in improving decision-making, assisting with crew self-reliance, providing mental health support, and developing personalized countermeasures for astronauts. Brain-computer interface technologies can be deployed to improve astronauts’ cognitive and operational capabilities. Other biotechnologies, such as new wearable devices and portable noninvasive monitoring equipment, 3D bioprinting, innovative drug delivery systems in long-acting drug delivery solutions, the use of induced pluripotent stem cells and organoids in regeneration, repair, and treatment, as well as microbial therapy in space, can be considered for optimizing the well-being of astronauts during long-term spaceflight.

Third, it is necessary to expedite the establishment of a biobank and datasets for Chinese astronauts to facilitate more original scientific findings and innovative discoveries in space science and enhance China’s discourse power in the field of international space medicine.

Fourth, TCM has unique advantages in providing comprehensive medical care for astronauts. The concept of “prevention is better than cure” is embodied in the application of TCM, and the application of TCM in space medicine has been ongoing [7]. In the upcoming lunar and Mars exploration flights, integrating TCM diagnostic methods with AI devices or integrating noninvasive TCM therapies with wearable devices have broad application prospects in maintaining the overall health of astronauts confined to limited space environments.

Finally, as space tourism becomes a reality, space medicine will need to evolve to cater to a broader range of individuals, including those without the rigorous training of professional astronauts. The development of medical protocols for space tourists, who may have preexisting health conditions or be of varying ages and fitness levels, is essential for ensuring their safety during space travel.

## Data Availability

Not applicable.

## Abbreviations

3D: Three-dimensional
AI: Artificial intelligence
CSS: Chinese Space Station
ISS: International Space Station
NASA: National Aeronautics and Space Administration
SOMA: Space Omics and Medical Atlas
TCM: Traditional Chinese medicine
